# Unveiling the efficacy of repetitive transcranial magnetic stimulation in Parkinson’s disease: A comprehensive review of systematic analyses

**DOI:** 10.1371/journal.pone.0313420

**Published:** 2025-01-06

**Authors:** Lingwen Zhang, Yanhong Jiang, Wenhui Fan, Hua Xue

**Affiliations:** 1 Department of Neurology, The Sixth People’s Hospital of Chengdu, Chengdu, Sichuan, China; 2 Department of Neurology, Sichuan Taikang Hospital, Chengdu, Sichuan, China; University of Catania: Universita degli Studi di Catania, ITALY

## Abstract

**Background:**

Many systematic reviews (SRs) have reported the repetitive transcranial magnetic stimulation (rTMS) for Parkinson’ s disease (PD), but the quality of the evidence is unclear. The aim of this study was to summarize the evidence provided by SRs on the effect of rTMS on PD.

**Methods:**

A comprehensive search for SRs published from the establishment of the library to March 1, 2024, was conducted in PubMed, EMBASE, Cochrane Library, Web of Science, CNKI, VIP and Wanfang databases. The A Measurement Tool to Assess Systematic Reviews 2 (AMSTAR-2), the Risk of Bias for Systematic Reviews (ROBIS), and Grading of Recommendations, Assessment, Development, and Evaluation (GRADE) tool were used to evaluate the methodology quality, risk of bias and evidence quality of SRs, respectively.

**Results:**

We identified 16 SRs. According to the results of the AMSTAR-2, 12.5% (2/16) of the SRs rated as high quality, 43.75% (7/16) rated as low quality, and 43.75% (7/16) rated as very low quality. Based on the ROBIS tool, 6 (37.5%) SRs had low risk of bias. The GRADE results suggested that 16.13% (10/62) of the evidence was of moderate quality, 33.87% (21/62) of the evidence was of low quality and 50% (31/62) of the evidence was of very low quality. Moderate-quality results show that rTMS can improve PD motor symptoms.

**Conclusions:**

Here we show that rTMS can improve the motor symptoms of PD, but its effectiveness in treating non-motor symptoms of PD is inconsistent. Due to the methodological limitations and diversity in study designs, future studies should focus on addressing these issues by providing thorough methodological details, standardizing rTMS protocols, evaluating side effects, and comparing with other treatments.

## 1. Introduction

Parkinson’s disease (PD) is the second most prevalent neurodegenerative disorder globally, affecting over 10 million individuals worldwide, ranking only behind Alzheimer’s disease [1]. PD presents with a range of clinical symptoms, including both motor and non-motor manifestations. Motor symptoms encompass bradykinesia, resting tremor, muscle stiffness, postural instability, among others, while non-motor symptoms consist of sleep disturbances, olfactory dysfunction, autonomic nervous system dysfunction, cognitive impairment, and psychiatric symptoms [2]. The main characteristic of PD is the loss of dopaminergic neurons in the substantia nigra. The death of these neurons leads to a decrease in dopamine levels in the brain, which in turn affects motor control and neurotransmission, leading to the motor symptoms of PD [3–5]. The pathogenesis of PD is intricate and involves multiple biological processes including oxidative stress, reduced antioxidant capacity, excitotoxicity, mitochondrial dysfunction, proteasome dysfunction, apoptosis, lysosomal dysfunction and impaired autophagy [6]. The treatment for PD consist of drug treatment and non-drug treatment. Drug treatment is typically the initial approach for managing motor symptoms and remains a key component throughout the course of PD treatment. However, prolonged use of these medications can result in motor complications (symptom fluctuations and dyskinesias) and non-motor complications (impulse control disorders), significantly impacting the patient’s quality of life and posing challenges for clinical management [7, 8]. Levodopa is the most frequently prescribed medication for the treatment of Parkinson’s disease; however, it may lead to side effects including involuntary movements, nightmares, orthostatic hypotension, constipation, nausea, and drowsiness [7]. Similarly, dopamine agonists can result in side effects such as drowsiness, nausea, orthostatic hypotension, obsessive-compulsive behavior, impulsivity, and hallucinations [8]. In cases where drug treatment proves ineffective or leads to severe side effects, surgical interventions may be considered. One such option is deep brain stimulation (DBS), where electrodes are implanted and adjusted via an external regulator to target specific brain areas and alleviate motor symptoms of PD. It is important to note that while DBS can be effective, it also carries risks and potential complications associated with surgery [4, 5].

Transcranial magnetic stimulation (TMS) is a physical method developed by Barker et al. to modulate brain function [9]. The primary mechanism involves the generation of a magnetic field by a coil during TMS treatment, which induces an electric field in the cortex [10, 11]. This electric field can alter the activity of voltage-gated ion channels in cell membranes, modify the membrane potential of cortical nerve cells, elicit induced currents, influence brain metabolism and neural electrical activity, enhance motor function, and alleviate symptoms such as depression and insomnia [10, 11]. Transcranial magnetic stimulation encompasses various stimulation modes, including repetitive transcranial magnetic stimulation (rTMS) and theta burst stimulation (TBS) [12]. rTMS modulates neural activity in the cerebral cortex by delivering a series of equally spaced magnetic field pulses continuously. Specifically, low-frequency rTMS (LF-rTMS, with a frequency between 0–2 Hz) can effectively inhibit cortical excitability, while high-frequency rTMS (HF-rTMS, with a frequency greater than 5 Hz) can enhance cortical excitability [13, 14]. Recent research highlights the significant effects of rTMS on brain structure and function. For instance, experimental observations indicate that rTMS can markedly increase dendritic complexity in the prefrontal cortex and primary motor cortex of mice, a change associated with the remodeling of neural circuits and the adjustment of intracortical connectivity [15, 16]. This intracortical rearrangement induced by rTMS may play a crucial role in ameliorating neurological dysfunction and promoting neural plasticity. Furthermore, intriguingly, in addition to inducing structural remodeling in the prefrontal cortex, HF-rTMS has also been found to exhibit anti-epileptiform activity [16]. This suggests that HF-rTMS not only modifies the activity patterns of neural networks but may also possess potential therapeutic effects for certain neurological disorders. In 1994, Pascual-Leone et al. pioneered the use of repetitive transcranial magnetic stimulation (rTMS) for treating motor symptoms in PD [17]. The parameters for rTMS treatment are intricate, encompassing aspects like stimulation site, intensity, frequency, pulse number, and treatment duration [18]. rTMS, as a novel therapeutic approach, offers advantages such as being painless, non-invasive, easy to administer, and safe [19]. Recent research has shown promising outcomes of rTMS in addressing freezing of gait symptoms in PD patients [20, 21]. A randomized controlled trial involving 46 participants demonstrated significant antidepressant effects of high-frequency rTMS applied to the left dorsolateral prefrontal cortex (DLPFC) region, and rTMS exhibited notable therapeutic benefits for PD motor symptoms [22]. Another study explored the potential therapeutic advantages of rTMS and other non-invasive stimulation methods, such as transcranial direct current stimulation, in managing depressive and non-motor symptoms in PD patients [23]. Findings indicate that rTMS has a positive impact on reducing depressive symptoms and cognitive impairment in individuals with PD.

A large number of systematic reviews (SRs) have summarized the efficacy and safety of rTMS for PD. SRs are considered the most important high-quality and reliable information in evidence-based medicine, it is important to note that not all systematic reviews are reliable. In fact, low-quality SRs have the potential to mislead clinical decisions. Given the wide variation in the quality of evidence across SRs in terms of methodological quality, risk of bias, and evidence quality, a thorough summary and critical assessment of relevant SRs is essential. This overview employs the Assessment Tool for Systematic Reviews 2 (AMSTAR-2), Risk of Bias for Systematic Reviews (ROBIS), and Grading of Recommendations, Assessment, Development, and Evaluation (GRADE) to comprehensively evaluate the SRs focusing on rTMS for PD [24–26]. The main objective of this review is to rigorously evaluate the quality of pertinent systematic reviews and provide an objective and comprehensive assessment of the efficacy and safety of rTMS in treating PD.

## 2. Methods

### 2.1 Data sources and search strategy

The systematic reviews of repetitive transcranial magnetic stimulation in the treatment of Parkinson ’s disease were collected by searching PubMed, EMBASE, Cochrane Library, Web of Science, CNKI, VIP and Wanfang databases. The time range of all searches is from the creation of the database to March 1, 2024. The search terms included “Parkinson’ s disease”, “repetitive transcranial magnetic stimulation”, “transcranial magnetic stimulation”, “meta-analysis”, “systematic evaluation”, “systematic review”. In addition, we also manually searched reviews related to repetitive transcranial magnetic stimulation and Parkinson ’s disease. We use Boolean logic to formulate a retrieval formula, which is applicable to all databases. The search strategy is shown in S1 Table.

### 2.2 Eligibility criteria

The following inclusion criteria were used to identify eligible studies: (1) study types: published Meta-analysis/systematic review of the literature on rTMS for PD, limited to Chinese and English; (2) participants: patients with PD and were diagnosed according to any internationally recognized clinical guidelines [4]; (3) intervention: the experimental group was treated with rTMS, and the treatment parameters such as frequency and treatment site were not limited, or rTMS combined with other treatments, including PD conventional drug treatment, acupuncture and moxibustion, etc. The control group was treated with drugs, sham stimulation or blank control; (4) outcomes: reviews that assessed the motor (such as UPDRS III, walking performance, timed up-and-go test, etc.) and non-motor symptoms (such as depression, cognitive impairment, etc.) of PD as the main outcome measures were considered eligible. Measures of quality of life and activities of daily living were included if they were relevant to the assessment of PD symptoms.

Exclusion criteria: (1) studies without complete data, such as conference abstracts, letters or comments; (2) network meta analysis or indirect comparison; (3) not rTMS.

### 2.3 Data extraction and management

Two authors (LW. Z and YH.J) independently conducted literature search and data extraction, and a third author (H.X) made the decision in case of disputes. Import the retrieved systematic reviews into Endnote, delete duplicate content, and select literature that may meet the criteria by reading the title and abstract. Systematic review that met the criteria were ultimately included by reading the full text. Information was extracted from all included systematic reviews. The extracted information included: authors, number of studies, number of samples, treatment group, control group, rTMS basic information, risk assessment tools, adverse reactions, outcomes, and main conclusions.

### 2.4 Quality assessment

We used A Measurement Tool to Assess Systematic Reviews 2 (AMSTAR 2) to evaluate the methodological quality of SRs [24]. The tool includes a total of 16 items, of which items 2, 4, 7, 9, 11, 13, and 15 are considered critical items, and the rest are non-critical items. Each item is described with “Yes” and “No”. If there are no defects or only one non-critical item defect, the methodological quality is rated “High” and the conclusion of SRs is accurate and comprehensive. If there is more than one non-critical item defect but no critical item defect, the methodological quality is rated as “Moderate” and the conclusion of the SRs is accurate. If there is one critical item defect, the methodological quality is rated as “Low” and the conclusion of the systematic review is low. If more than one critical entry is rated as “No”, the methodological quality is “Very low” and the system evaluation decisions are inaccurate and incomplete. Two authors independently assessed the methodological quality of included SRs.

We used the Risk of Bias for Systematic Reviews (ROBIS) tool to assess the risk of bias (RoB) for SRs [25]. The evaluation process is divided into three phases: (1) assessing relevance; (b) phase 2 included four domain (study eligibility criteria, identification and selection of studies, collection and study appraisal, synthesis and findings); (3) risk of bias in the review. Two authors independently classified the risk levels into “low risk”, “high risk” and “unclear risk” and any disagreements were adjudicated by a third author.

We used GRADE to assess the quality of the primary results of SRs [26]. Five key factors that affect the quality of evidence include: study limitations, inconsistency of results, indirectness of evidence, imprecision and reporting bias. The quality of evidence for SRs was rated as “High” (not downgraded), “Moderate” (downgraded one level), “Low” (downgraded two levels), and “Very low” (downgraded three or more levels).

## 3. Results

### 3.1 Literature retrieval results

**Fig 1:**
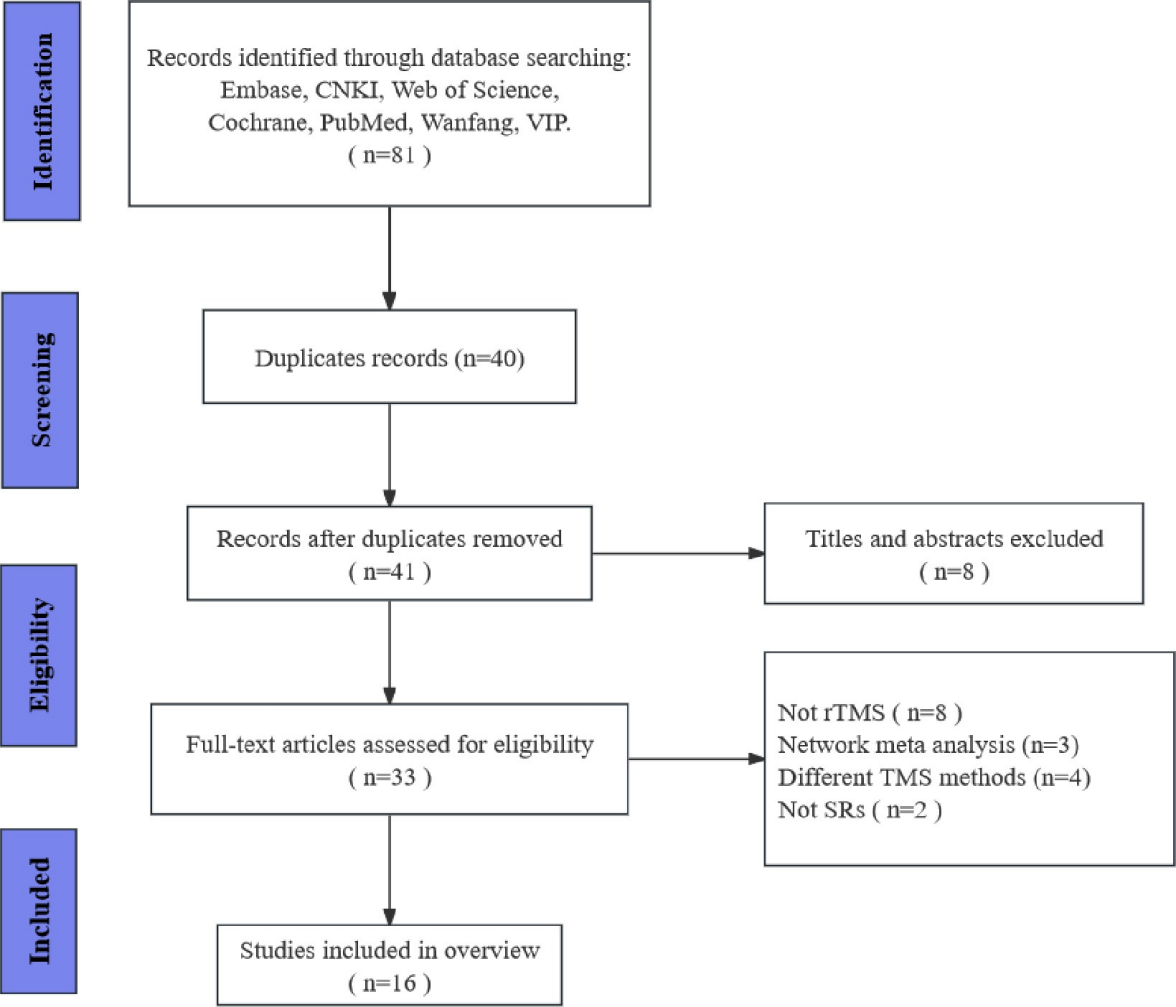
Flow chart of the literature search and study selection process.

Following the search strategy, a total of 81 systematic reviews (SRs) were identified. After excluding 40 duplicate papers through filtering and 8 papers based on title and abstract review, the remaining 33 SRs underwent full-text evaluation. Among these, 8 articles with interventions other than repetitive Transcranial Magnetic Stimulation (rTMS), 3 network meta-analyses, 4 articles comparing various TMS techniques, and 2 non-SRs were eliminated, resulting in a final inclusion of 16 articles. The literature screening process is shown in Fig 1.

### 3.2 Basic features of the included literature

The basic characteristics of the included 16 SRs are shown in Table 1 [27–42]. A total of 16 SRs met inclusion criteria, and the literature published period ranged from 2015 to 2022. The total number of included original studies was 260, with a maximum of 32 and a minimum of 5. Sample sizes ranged from 160 to 1084, with 3 SRs not reporting sample sizes [30, 31, 33]. The treatment in the control group was mainly PD routine treatment, routine treatment combined with sham stimulation, and only sham stimulation. The treatment of the experimental group was mainly rTMS combined with routine treatment, and a variety of rTMS. The basic characteristics of rTMS include a stimulation frequency ranging from 0.2 to 50 Hz, stimulation intensity varying from 70% to 120% of resting motor threshold (RMT), and a number of pulses ranging from 100 to 3000. Common stimulation sites are the left (or right) dorsolateral prefrontal cortex (lDLPFC or rDLPFC), left occipital cortex (LOC), inferior frontal gyrus (IFG), supplementary motor area (SMA), primary motor cortex (M1), premotor dorsal cortex (PMD), motor cortex (MC), and prefrontal cortex (PFC). For risk assessment, 12 SRs used the Cochrane risk of bias tool, 1 SR used the Jadad Quality Assessment Scale, 1SR used the Physiotherapy Evidence Database (PEDro) scale, 1 SR used the Oxford Center for Evidence Based Medicine levels, and one did not mention bias assessment. Overall, while many researchers suggest that rTMS shows promise in treating PD, further high-quality studies are needed to confirm these findings.

**Table 1:** Characteristics of the included systematic reviews.

Included studies	Number of studies	Participants	Experimental Intervention	Control Intervention	Basic features of rTMS	Risk assessment tools	Adverse effects	Outcomes	Main conclusion
Chen et al. 2021 [27]	12	511	rTMS; rTMS + routine treatment; rTMS + fluoxetine/setraline + routine treatment.	routine treatment; sham stimulation + routine treatment; routine treatment + fluoxetine/setraline.	0.5/1/5/10/15 Hz; lDLPFC/rDLPFC/Bilateral M1; 90%/100%/110% /120% RMT; 600/800/1200/1740/2000 pulses/day; 10/14 /28 consecutive days.	Cochrane risk of bias tool	Headache and neck pain	HAMD, UPDRS III	rTMS can safely alleviate depression and motor symptoms in PD at least for a short period.
Chung et al. 2016 [28]	22	555	rTMS; rTMS + routine treatment	routine treatment; sham stimulation + routine treatment.	0.5/1/5/10/50 Hz; lDLPFC/rDLPFC/Bilateral M1; 70%/80%/90%/100%/110% RMT or AMT; 100/450/900/1000/1200/1500/1800/3000 pulses/day; 4/6/10/14/28 consecutive days.	Cochrane risk of bias tool	Not reported	UPDRS III, Walking performance, Upper limb function	The pooled evidence suggests that rTMS improves upper limb function in the short term, walking performance and UPDRS III in the short and long-terms in PD sufferers.
Deng et al. 2022 [29]	16	419	rTMS	sham stimulation	1/5/10/25/50 Hz; lDLPFC/rDLPFC/Bilateral M1/SMA; 80%/90%/100%/110% RMT; 600/720/1000/1200/1700 pulses/day.	Cochrane risk of bias tool	Not reported	FOG-Q, walking time, TUG, MoCA,	rTMS showed a beneficial effect on manage freezing of gait and cognitive dysfunction in parkinsonism. However, the optimal rTMS protocol has not been determined and further high-quality studies are needed.
Goodwill et al. 2017 [30]	15	Not reported	rTMS	sham stimulation	0.2/5/10/25/50 Hz; lDLPFC/rDLPFC/Bilateral M1/SMA; 70%/80%/90%/100%/110% /120% RMT; 100/300/450/600/900/1300/1800/2000/2250 pulses/day; 3/10/14/28 consecutive days.	Cochrane risk of bias tool	Not reported	UPDRS III, gait performance, hand movement, cognitive function	rTMS not improved cognition.
Han et al. 2015 [31]	13	Not reported	rTMS + routine treatment	routine treatment; sham stimulation + routine treatment;	0.2/1/5/10/25/50 Hz; lDLPFC/rDLPFC/Bilateral M1/SMA; 80%/90%/100%/110% /120% RMT; 60/100/450/1000/1200/1600/2000 pulses/day; 1/2/3/ months, 2/4/16 weeks.	Cochrane risk of bias tool	Not reported	UPDRS III	rTMS can effectively relieve PD patients’ dyskinesia.
He et al. 2020 [32]	12	230	rTMS; rTMS + routine treatment	routine treatment; sham stimulation + routine treatment.	5/10/20/25/50 Hz; LOC/IFG/lDLPFC/Bilateral M1; 80%/90%/100%/110% RMT; 500/600/1200/1350/4000 pulses/day; 3/10 consecutive days, 2/4 weeks.	Cochrane risk of bias tool	Not reported	Global cognition, Executive function, Attention and working memory	Based on a limited number of studies, rTMS fails to improve cognition in PD.
Li et al. 2020 [33]	28	Not reported	rTMS; rTMS + routine treatment	routine treatment; sham stimulation + routine treatment.	0.2/0.5/1/5/10/15/50 Hz; lDLPFC/rDLPFC/Bilateral M1/SMA; 80%/90%/100%/110%/120% RMT; 600/1000/1350/1800/3000/3750 pulses/day; 3/10/12 consecutive days, 1/2/4/8 weeks.	None	Not reported	Motor function, depression	The rTMS showed significant therapeutic effects on motor in PD. High frequency rTMS showed a significant positive antidepressive effect in PD only over DLPFC.
Li et al. 2022 [34]	32	1048	rTMS; rTMS + routine treatment	routine treatment; sham stimulation + routine treatment.	0.2/1/5/10/20/25 Hz; lDLPFC/Bilateral M1/SMA.	Cochrane risk of bias tool	Not reported	UPDRS III, walking time, FOG-Q, TUG.	rTMS therapy is an effective treatment for motor symptoms of PD and the individualized stimulation protocols for different symptoms would further improve its clinical efficacy.
Nehra et al. 2020 [35]	5	160	rTMS; rTMS + routine treatment	routine treatment; sham stimulation + routine treatment.	5~15 Hz; DLPFC/M1/SMA; 600~6000 pulses/day; > 10 days.	Cochrane risk of bias tool	Headache and neck pain	Quality of Life	The efficacy of rTMS as an adjunct intervention to enhance quality of life of PD patients is uncertain due to dire lack of research in this area.
Qin et al. 2018 [36]	9	332	rTMS; rTMS + routine treatment; rTMS + fluoxetine/paroxetine + routine treatment.	routine treatment; sham stimulation + routine treatment; routine treatment + fluoxetine/paroxetine.	5/10/15 Hz; lDLPFC/rDLPFC/Bilateral M1; 90%/100%/110%/120% RMT.	Cochrane risk of bias tool	Not reported	Depressive symptoms, UPDRS III.	This meta-analysis provides some evidence that in patients with PD with depression, HF-rTMS may lead to improvement in motor function but not in depression compared with sham-rTMS or SSRIs.
Wagle et al. 2016 [37]	21	671	rTMS; rTMS + routine treatment.	routine treatment; sham stimulation + routine treatment.	0.2/0.5/1/5/10/15/50 Hz; DLPFC/Bilateral M1/PMD/SMA; 80%/90%/100%/110% RMT; 6/10 days, 2/4 weeks.	Oxford Centre for Evidence Based Medicine levels	Not reported	UPDRS III	rTMS therapy in patients with Parkinson disease results in mild-to-moderate motor improvements and has the potential to be used as an adjunct therapy for the treatment of Parkinson disease.
Xie et al. 2020 [38]	14	298	rTMS; rTMS + routine treatment.	routine treatment; sham stimulation + routine treatment.	1/5/10/25/50 Hz; PFC/M1/DLPFC; 80%/90%/100%/110%/120% RMT; 3/6/10 days, 2/3/ 4 weeks.	the Physiotherapy Evidence Database (PEDro) scale	Not reported	Walking time, Freezing of gait, TUG	The results of the meta-analysis propose the favorable effect of rTMS on walking performance in the short-term but not in the long term in individuals with PD.
Yang et al. 2018 [39]	23	765	rTMS; rTMS + routine treatment.	routine treatment; sham stimulation + routine treatment.	0.2/1/5/10/25/50 Hz; DLPFC/ M1/SMA/PMD; 80%/90%/100%/110% RMT; 100/450/900/1000/1800/2000/3000 pulses/day.	Cochrane risk of bias tool	Mild and transient headache and neck pain.	UPDRS III	In conclusion, multi-session of HF-rTMS over the M1 (especially bilateral M1) with a total of 18,000–20,000 pulses appears to be the optimal parameters for motor improvement of PD.
Zhang et al. 2022 [40]	14	469	rTMS; rTMS + routine treatment.	routine treatment; sham stimulation + routine treatment.	1/5/10/25/50 Hz; DLPFC/ M1/SMA/PMD; 80%/90%/100%/110% RMT.	Cochrane risk of bias tool	Not reported	UPDRS III, MMSE, MoCA, DRS-2, BDI, HAMD, MADRS.	The findings suggest that rTMS could be used as a possible adjuvant therapy for PD mainly to improve motor symptoms, but could have potential efficacy on depressive symptoms of PD.
Zhao et al. 2015 [41]	16	455	rTMS; rTMS + routine treatment.	routine treatment; sham stimulation + routine treatment.	0.2/0.5/1/5/15/25/50 Hz; DLPFC/ M1; 60%/90%/100%/110% /120% RMT.	Jadad Quality Assessment Scale	Not reported	UPDRS III, ADL, MMSE.	Repetitive transcranial magnetic stimulation therapy can ameliorate partial symptoms of Parkinson’s disease for enhancing the quality of life, however, the improvement for mental disability was not found.
Zhu et al. 2015 [42]	8	319	rTMS; rTMS + routine treatment.	routine treatment; sham stimulation + routine treatment.	0.2/1 Hz; DLPFC/Bilateral M1/SMA; 90%/110%/120% RMT.	Cochrane risk of bias tool	Not reported	UPDRS III	Low-frequency rTMS had a significant effect on motor signs in PD. As the number of RCTs and PD patients included here was limited, further large-scale multi-center RCTs were required to validate our conclusions

PD, Parkinson’ s Disease; rTMS, repetitive transcranial magnetic stimulation; lDLPFC or rDLPFC, left (or right) dorsolateral prefrontal cortex; LOC, Left occipital cortex; IFG: the inferior frontal gyrus; SMA, the supplementary motor area; RMT, resting motor threshold; AMT, active motor threshold; M1, primary motor cortex; PMD, premotor dorsal cortex; MC, motor cortex; PFC, prefrontal cortex; UPDRS III, Unified Parkinson’ s Disease Rating Scale III; HAMD, Hamilton Depression Scale; FOG-Q, freezing of gait questionnaire; TUG, timed up-and-go test; MMSE, the Mini-Mental State Exam; MoCA, Montreal Cognitive Assessent; DRS-2, the Mattis Dementia Rating Scale-2; BDI, the Beck Depression Inventory; MADRS, Montgomery Asberg Depression Rating Scale; SSRIs, selective serotonin reuptake inhibitors.

### 3.3 Evaluation results of methodological quality

**Fig 2:**
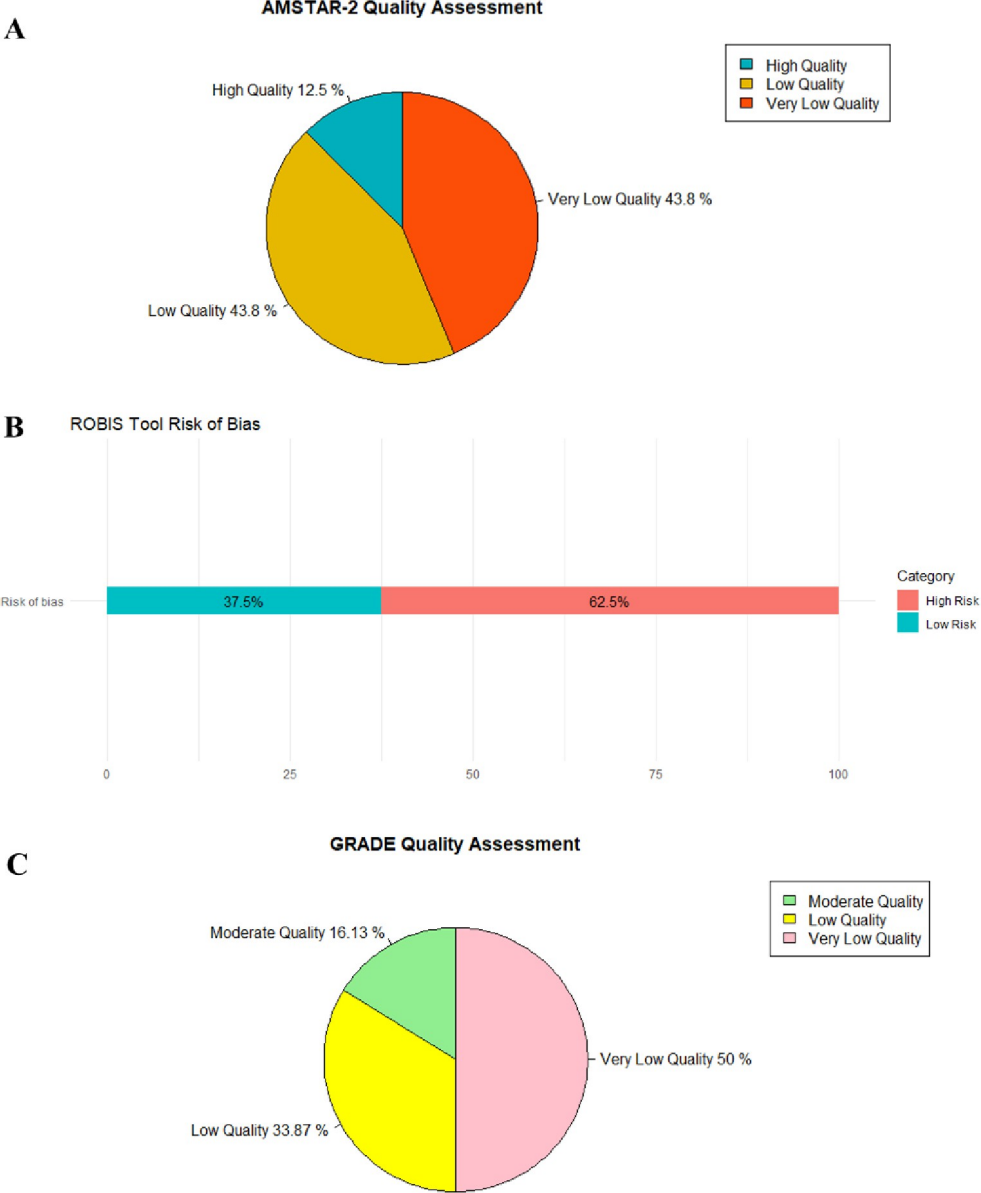
Methodological quality, risk of bias, and evidence quality of included systematic reviews. (A) AMSTAR 2; (B) Risk of bias; (C) evidence quality.

Table 2 displays the results of evaluating methodological quality using the AMSTAR 2 tool. Of the 16 SRs, 2 (12.5%) were rated as high quality (29,40), 7 (43.75%) were rated as low quality [27, 28, 32, 34, 38, 39, 41], and 7 (43.75%) were rated as very low quality [30, 31, 33, 35–37, 42] (Fig 2A). Among the critical items, item 2 has a compliance rate of 18.75%, item 4 has a compliance rate of 68.75%, item 7 has a compliance rate of 75%, item 9 has a compliance rate of 75%, item 11 has a compliance rate of 68.75%, item 13 has a compliance rate of 81.25%, and item 15 has a compliance rate of 75%, thus most of the SRs are deficient in the critical items. Among the non-critical items, items 1, 3, 10, and 16 all met the standards, the compliance rate for items 5, 6, and 8 was 68.75%, and the compliance rate for items 12 and 14 was 62.5%.

**Table 2:** Methodological quality of included systematic reviews.

Included Studies	AMSTAR 2																Overall Quality
	Q 1	Q 2*	Q 3	Q 4*	Q 5	Q 6	Q 7*	Q 8	Q 9*	Q 10	Q 11*	Q 12	Q 13*	Q 14	Q 15*	Q 16	
Chen et al. 2021 [27]	Y	N	Y	Y	Y	Y	Y	Y	Y	Y	Y	Y	Y	Y	Y	Y	Low
Chung et al. 2016 [28]	Y	N	Y	Y	N	N	Y	N	Y	Y	Y	N	Y	N	Y	Y	Low
Deng et al. 2022 [29]	Y	Y	Y	Y	Y	Y	Y	Y	Y	Y	Y	Y	Y	Y	Y	Y	High
Goodwill et al. 2017 [30]	Y	N	Y	Y	N	N	Y	Y	N	Y	N	Y	Y	Y	N	Y	Very low
Han et al. 2015 [31]	Y	N	Y	N	N	N	N	N	N	Y	Y	N	N	Y	Y	Y	Very low
He et al. 2020 [32]	Y	N	Y	Y	Y	Y	Y	Y	Y	Y	Y	Y	Y	Y	Y	Y	Low
Li et al. 2020 [33]	Y	N	Y	N	Y	Y	N	N	N	Y	N	Y	N	Y	N	Y	Very low
Li et al. 2022 [34]	Y	N	Y	Y	Y	Y	Y	Y	Y	Y	Y	Y	Y	Y	Y	Y	Low
Nehra et al. 2020 [35]	Y	N	Y	N	Y	Y	N	Y	N	Y	N	N	N	N	N	Y	Very low
Qin et al. 2018 [36]	Y	Y	Y	N	Y	Y	Y	Y	Y	Y	N	N	Y	Y	N	Y	Very low
Wagle et al. 2016 [37]	Y	N	Y	N	N	N	N	Y	Y	Y	Y	N	Y	Y	Y	Y	Very low
Xie et al. 2020 [38]	Y	N	Y	Y	Y	Y	Y	Y	Y	Y	Y	Y	Y	N	Y	Y	Low
Yang et al. 2018 [39]	Y	N	Y	Y	Y	Y	Y	Y	Y	Y	Y	Y	Y	N	Y	Y	Low
Zhang et al. 2022 [40]	Y	Y	Y	Y	Y	Y	Y	Y	Y	Y	Y	Y	Y	Y	Y	Y	High
Zhao et al. 2015 [41]	Y	N	Y	Y	Y	Y	Y	N	Y	Y	Y	Y	Y	N	Y	Y	Low
Zhu et al. 2015 [42]	Y	N	Y	Y	N	N	Y	N	Y	Y	N	N	Y	N	Y	Y	Very low
Y / total (%)	100	18.75	100	68.75	68.75	68.75	75	68.75	75	100	68.75	62.5	81.25	62.5	75	100	

Y, yes; N, no. Note: The key items of the AMSTAR 2. H: represents the ranking of quality as high; M: represents the ranking of quality as moderate; L: represents the ranking of quality as low; CL: represents the ranking of quality as critically low. Q1: did the research questions and inclusion criteria for the review include the components of PICO? Q2: did the report of the review contain an explicit statement that the review methods were established prior to the conduct of the review and did the report justify any significant deviations from the protocol? Q3: did the review authors explain their selection of the study designs for inclusion in the review? Q4: did the review authors use a comprehensive literature search strategy? Q5: did the review authors perform study selection in duplicate? Q6: did the review authors perform data extraction in duplicate? Q7: did the review authors provide a list of excluded studies and justify the exclusions? Q8: did the review authors describe the included studies in adequate detail? Q9: did the review authors use a satisfactory technique for assessing the risk of bias (RoB) in individual studies that were included in the review? Q10: did the review authors report on the sources of funding for the studies included in the review? Q11: if a meta-analysis was performed, did the review authors use appropriate methods for the statistical combination of results? Q12: if a meta-analysis was performed, did the review authors assess the potential impact of RoB in individual studies on the results of the meta-analysis or another evidence synthesis? Q13: did the review authors account for RoB in primary studies when interpreting/ discussing the results of the review? Q14: did the review authors provide a satisfactory explanation for, and discussion of, any heterogeneity observed in the results of the review? Q15: if they performed quantitative synthesis did the review authors carry out an adequate investigation of publication bias (small study bias) and discuss its likely impact on the results of the review? Q16: did the review authors report any potential sources of conflict of interest, including any funding they received for conducting the review? Abbreviations: AMSTAR 2, Assessment of Multiple Systematic Reviews 2; N, no; PY, partial yes; Y, yes.

### 3.4 Results of ROBIS evaluation

In the first stage of ROBIS, the relevance of the study topic was assessed, and all systematic reviews (SRs) were deemed to have a low risk of bias. Domain 1 evaluated study eligibility criteria, with all 11 SRs rated as low risk of bias [27–30, 32, 34, 38–42]. Domain 2 looked at the identification and selection of studies, revealing that 9 SRs had a low risk of bias [27–30, 32, 34, 38–42]. Domain 3 focused on the collection and evaluation of studies, finding that 11 SRs were at low risk of bias [27–30, 32, 34, 36–38, 40–42]. Domain 4 examined synthesis and findings, showing that 6 out of 16 SRs were rated as low risk of bias [27, 29, 34, 38–40]. In the final stage, the overall risk of bias of the reviews was considered, and 6 (37.5%) SRs were found to be at low risk of bias [27, 29, 34, 38–40]. Refer to Table 3 and Fig 2B for more detailed information.

**Table 3:** Risk of bias of the included systematic reviews.

Included Studies	Phase 1	Phase 2				Phase 3
	Assessing relevance	Domain 1: study eligibility criteria	Domain 2: identification and selection of studies	Domain 3: collection and study appraisal	Domain 4: synthesis and findings	Risk of bias in the review
Chen et al. 2021 [27]	low risk	low risk	low risk	low risk	low risk	low risk
Chung et al. 2016 [28]	low risk	low risk	low risk	low risk	high risk	high risk
Deng et al. 2022 [29]	low risk	low risk	low risk	low risk	low risk	low risk
Goodwill et al. 2017 [30]	low risk	low risk	low risk	high risk	high risk	high risk
Han et al. 2015 [31]	low risk	high risk	high risk	high risk	high risk	high risk
He et al. 2020 [32]	low risk	low risk	low risk	low risk	high risk	high risk
Li et al. 2020 [33]	low risk	high risk	high risk	high risk	high risk	high risk
Li et al. 2022 [34]	low risk	low risk	low risk	low risk	low risk	low risk
Nehra et al. 2020 [35]	low risk	high risk	high risk	high risk	high risk	high risk
Qin et al. 2018 [36]	low risk	high risk	high risk	low risk	high risk	high risk
Wagle et al. 2016 [37]	low risk	high risk	high risk	low risk	high risk	high risk
Xie et al. 2020 [38]	low risk	low risk	low risk	low risk	low risk	low risk
Yang et al. 2018 [39]	low risk	low risk	low risk	high risk	low risk	low risk
Zhang et al. 2022 [40]	low risk	low risk	low risk	low risk	low risk	low risk
Zhao et al. 2015 [41]	low risk	low risk	high risk	low risk	high risk	high risk
Zhu et al. 2015 [42]	low risk	low risk	high risk	low risk	high risk	high risk

### 3.5 Evidence quality level evaluation results

Using the GRADE system, 16 papers were analyzed for 62 outcome indicators. The findings revealed that there was no high quality evidence present in the SRs. Moderate quality evidence was found in 10 outcomes (16.13%), while low quality evidence was present in 21 outcomes (33.87%), and very low quality evidence in 31 outcomes (50%). Overall, the quality of evidence was deemed to be generally low. This was attributed to several factors: (1) Serious flaws in blinding, allocation concealment, and randomization in the included literature, impacting the validity of research results; (2) Heterogeneity in outcome indicators affecting result reliability; and (3) Asymmetric forest plot suggesting potential publication bias. Further details on the evaluation of evidence quality can be found in Table 4 and Fig 2C.

**Table 4:** Results of evidence quality with GRADE.

Included Studies	Outcomes	Included Studies	Inconsistency	Indirectness	Imprecision	Publication bias	Relative effect (95% CI)	I^2^	P-value	Quality
Chen et al. 2021 [27]	Short-term effect: HAMD (9)	-1	0	0	0	-1	SMD -0.62 (-0.964, -0.278)	62.2%	0.007	Low
	Long-term effect: HAMD (4)	-1	0	0	-1	-1	SMD -0.56 (-1.421, 0.301)	81.0%	0.203	Very low
	Short-term effect: UPDRS III (7)	-1	0	0	-1	-1	WMD -2.62 (-4.183, -1.051)	0	0.001	Very low
	Long-term effect: UPDRS III (4)	-1	0	0	-1	-1	WMD—1.63 (-5.03, 1.78)	0	0.350	Very low
Chung et al. 2016 [28]	Short-term effect: UPDRS III (20)	-1	0	0	-1	0	MD 0.31 (0.11, 0.51)	32.3%	0.003	Low
	Long-term effect: UPDRS III (8)	-1	0	0	-1	0	MD 0.54 (0.18, 0.89)	59.3%	0.003	Low
	Short-term effect: Walking performance (9)	-1	0	-1	-1	-1	MD 0.61 (0.05, 1.17)	77.4%	0.03	Very low
	Long-term effect: Walking performance (5)	-1	0	-1	-1	-1	MD 0.89 (0.10, 0.68)	80.4%	0.03	Very low
	Short-term effect: Upper limb function (8)	-1	0	0	-1	0	MD 0.40 (0.11, 0.68)	0	0.007	Low
	Long-term effect: Upper limb function (3)	-1	0	0	-1	-1	MD 0.53 (-0.09, 1.14)	NR	0.09	Very low
Deng et al. 2022 [29]	Short-term effect: FOG-Q (5)	-1	0	0	0	0	WMD -0.925 (-1.642, -0.209)	0	0.011	Moderate
	Long-term effect: FOG-Q (7)	-1	0	0	0	0	WMD -2.120 (-2.751, -1.489)	15.4%	< 0.0001	Moderate
	Short-term effect: walking time (3)	-1	0	-1	0	-1	WMD -0.456 (-0.793, -0.119)	0	0.008	Very low
	Long-term effect: walking time (2)	-1	0	-1	0	-1	WMD -0.526 (-0.885, -0.167)	0	0.004	Very low
	Short-term effect: TUG test (4)	-1	0	0	0	0	WMD -1.064 (-1.555, -0.572)	0	< 0.0001	Moderate
	Long-term effect: TUG test (5)	-1	0	0	0	0	WMD -1.097 (-1.422, -0.772)	0	< 0.0001	Moderate
	MoCA (5)	-1	-1	0	0	-1	WMD 2.67 (0.513, 4.827)	75.9%	0.015	Very low
Goodwill et al. 2017 [30]	Gait performance (7)	-1	0	-1	0	-1	SMD 0.705 (0.012, 1.398)	NR	0.046	Very low
	UPDRS III (20)	-1	0	0	0	-1	SMD 0.371 (0.084, 0.659)	NR	0.011	Low
	Hand movements (6)	-1	0	-1	0	-1	SMD 0.538 (-0.227, 1.353)	NR	0.195	Very low
	Cognitive function (5)	-1	0	0	-1	-1	SMD 0.271 (-0.43,0.974)	NR	0.982	Very low
Han et al. 2015 [31]	UPDRS III (13)	-1	0	0	0	-1	WMD -3.97 (-5.79, -2.16)	50%	< 0.01	Low
	LF-rTMS: UPDRS III (8)	-1	-1	0	0	0	WMD -2.66 (-4.17, -1.15)	39%	< 0.01	Low
	HF-rTMS: UPDRS III (6)	-1	-1	0	0	0	WMD -5.86 (-7.22, -4.50)	4%	< 0.01	Low
He et al. 2020 [32]	Short-term effect: global cognition (6)	-1	0	-1	-1	0	SMD -0.15 (-0.59, 0.29)	36.7%	0.162	Very low
	Long-term effect: global cognition (5)	-1	0	-1	-1	0	SMD 0.10 (0.44, 0.24)	0	0.595	Very low
	Short-term effect: executive function (13)	-1	-1	0	0	0	SMD 0.03 (0.21, 0.26)	0	0.993	Low
	Long-term effect: executive function (6)	-1	-1	0	0	0	SMD 0.13 (-0.19, 0.44)	0	0.803	Low
	Short-term effect: attention and wording memory (8)	-1	0	-1	-1	0	SMD 0.05 (0.25, 0.35)	0	0.990	Very low
	Long-term effect: attention and wording memory (3)	-1	0	-1	-1	0	SMD 0.02 (0.39, 0.42)	0	0.988	Very low
Li et al. 2020 [33]	Motor function (28)	-1	-1	0	-1	-1	MD 2.05 (1.57, 2.53)	93%	< 0.0001	Very low
	Depression (19)	-1	-1	0	-1	-1	MD 0.80 (0.31, 1.29)	89.1%	< 0.0001	Very low
Li et al. 2022 [34]	UPDRS III	-1	-1	0	0	0	SMD 0.64 (0.47, 0.80)	64%	<0.0001	Low
	Walking time	-1	0	0	0	-1	SMD 0.95 (0.61, 1.28)	64%	<0.0001	Low
	FOG-Q	-1	0	0	0	-1	SMD 0.39 (0.04, 0.73)	64%	<0.0001	Low
	TUG test	-1	0	0	0	-1	SMD 0.90 (0.34, 1.45)	64%	<0.0001	Low
Qin et al. 2018 [36]	Depressive symptoms (11)	-1	-1	0	0	-1	SMD -0.17 (-0.52, 0.18)	54%	0.34	Very low
	HF-rTMS: depressive symptoms (6)	-1	-1	0	0	-1	SMD -0.33 (-0.83, 0.17)	60%	0.20	Very low
	UPDRS III (10)	-1	0	0	0	-1	SMD -2.70 (-4.51, 0.90)	0	0.003	Low
Wagle et al. 2016 [37]	UPDRS	-1	-1	0	0	-1	MD 3.3 (1.6, 5.0)	NR	0.005	Very low
	Short-term effect: UPDRS	-1	-1	0	0	-1	MD 3.4 (0.3, 6.6)	NR	0.04	Very low
	Long-term effect: UPDRS	-1	-1	0	0	-1	MD 4.1 (-0.15, 8.5)	NR	0.05	Very low
Xie et al. 2020 [38]	Walking time (9)	-1	0	0	0	0	SMD -0.30 (-0.57, -0.03)	24%	0.03	Moderate
	FOG-Q (4)	-1	-1	0	0	-1	SMD -0.81 (-1.68, 0.06)	71%	0.07	Very low
	TUG test (4)	-1	-1	0	0	-1	SMD -0.45 (-1.32, 0.41)	80%	0.30	Very low
Yang et al. 2018 [39]	UPDRS III (33)	-1	0	0	0	0	SMD 0.37 (0.24, 0.50)	29%	<0.0001	Moderate
	HF-rTMS: UPDRS III (21)	-1	0	0	0	0	SMD 0.48 (0.32, 0.64)	45%	<0.0001	Moderate
	LF-rTMS: UPDRS III (10)	-1	0	0	0	0	SMD 0.19 (-0.04, 0.42)	0	<0.0001	Moderate
Zhang et al. 2022 [40]	UPDRS III (17)	-1	0	0	0	0	SMD 0.51 (0.30, 0.71)	29%	<0.0001	Moderate
	HF-rTMS: UPDRS III (15)	-1	0	0	0	0	SMD 0.56 (0.34, 0.77)	29%	<0.0001	Moderate
	LF-rTMS: UPDRS III (2)	-1	0	0	0	-1	SMD 0.10 (-0.44, 0.64)	0	0.73	Low
	MMSE (2)	-1	-1	0	-1	-1	SMD 0.10 (-0.39, 0.58)	0	0.69	Very low
	MoCA (1)	-1	-1	0	-1	-1	SMD -0.75 (-1.37, -0.14)	NR	0.02	Very low
	DRS-2 (1)	-1	-1	0	-1	-1	SMD 0.19 (-0.39, 0.77)	NR	0.52	Very low
	BDI (3)	-1	0	0	0	-1	SMD 0.51 (0.12, 0.89)	0	0.01	Low
	HAMD (2)	-1	-1	0	-1	-1	SMD 0.18 (-0.25, 0.61)	10%	0.42	Very low
	MADRS (2)	-1	-1	0	-1	-1	SMD 0.55 (-0.31, 1.41)	63%	0.21	Very low
Zhao et al. 2015 [41]	HF-rTMS: UPDRS III (8)	-1	-1	0	0	-1	WMD -4.38 (-8.26, -0.50)	82%	0.003	Very low
	LF-rTMS: UPDRS III (5)	-1	0	0	0	-1	WMD -2.16 (-5.01, -0.69)	0	0.14	Low
	ADL (3)	-1	0	0	0	-1	WMD -3.74 (-4.66, -2.82)	0	<0.0001	Low
	MMSE (3)	-1	0	0	0	-1	WMD 0.26 (-0.66, -1.19)	0	0.58	Low
Zhu et al. 2015 [42]	UPDRS III (8)	-1	0	0	0	-1	WMD -0.40 (-0.73, -0.06)	47%	< 0.05	Low

−1, downgrade; 0, not downgrade; CL, critically low; L, low; M, moderate; H, high; UPDRS III, Unified Parkinson’ s Disease Rating Scale III; HAMD, Hamilton Depression Scale; FOG-Q, freezing of gait questionnaire; TUG, timed up-and-go test; MMSE, the Mini-Mental State Exam; MoCA, Montreal Cognitive Assessent; DRS-2, the Mattis Dementia Rating Scale-2; BDI, the Beck Depression Inventory; MADRS, Montgomery Asberg Depression Rating Scale.

### 3.6 Main outcome measures

Most SRs have focused on the efficacy and safety improvements of rTMS in patients with PD in the following areas: motor symptoms, depressive symptoms, cognitive function, ability to perform activities of daily living, and adverse effects.

#### 3.6.1 Motor symptom

Motor symptoms are the main clinical manifestations of PD. The 16 SRs selected Unified Parkinson’ s Disease Rating Scale III (UPDRS III), walking performance, gait performance, upper limb function, hand movements, walking time, freezing of gait questionnaire (FOG-Q) and timed up-and-go test (TUG) to evaluate motor function.

11 SRs compared the effect of rTMS in the treatment of PD using UPDRS / UPDRSIII scores [27, 28, 30, 31, 34, 36, 37, 39–42]. Most of the results showed that rTMS was more effective than conventional PD treatment or sham stimulation treatment. Two SRs studies showed no statistical difference in the long-term effect size of rTMS treatment compared with non-rTMS treatment in relieving motor symptoms [WMD = -1.63, 95% CI (-5.03, 1.78), P = 0.35; MD = 4.1, 95% CI (-0.15, 8.5), P = 0.05) [27, 37]. In addition, two SRs showed no difference between LF-rTMS treatment and control treatment in improving motor symptoms [WMD = 0.10, 95% CI (-0.44, 0.64), P = 0.73; WMD = -2.16, 95% CI (-5.01, -0.69), P = 0.14) [40, 41].

Chung et al evaluated walking performance and upper limb function, pooled estimates of effect of rTMS indicated significantly improved short-term upper limb function, short-term and long-term walking performance [27]. Three SRs used FOG-Q to evaluate the effect of rTMS in the treatment of PD, and the analysis of two SRs suggested that rTMS was effective in improving freezing of gait questionnaire scores [29, 34]. Xie et al.’ s analysis of SRs showed no significant difference in scores on the FOG-Q between rTMS and no intervention [SMD = -0.81, 95% CI (-1.68, 0.06), P = 0.07] [38]. Three SRs used walking time to evaluate the effect of rTMS in the treatment of PD. The results showed that compared with sham transcranial magnetic stimulation, rTMS improved walking time [29, 34, 38]. Three SRs assessed the TUG test in PD patients treated with rTMS. Two SRs reported that the rTMS-treated group spent significantly less time in the TUG test compared to the control group, while one SR found no significant difference between the two groups [SMD = -0.81, 95% CI (-1.68, 0.06), P = 0.07] [38]. In included SRs, 19.51% of studies did not find significant improvement in motor symptoms of PD when rTMS was compared with traditional treatment.

#### 3.6.2 Non-motor symptom

The included SRs selected the Hamilton Depression Scale (HAMD), the Mini-Mental State Exam (MMSE), the Montreal Cognitive Assessent (MoCA), the Mattis Dementia Rating Scale-2 (DRS-2), and the Beck Depression Inventory (BDI) to assess non-motor symptoms.

Four SRs evaluate the depressive symptoms of PD treated with rTMS. Research by Chen et al. indicated that rTMS can significantly improve depression in the short term [SMD = -0.62, 95% CI (-0.964, -0.278), P = 0.007], but cannot significantly improve depression scores in the long term [SMD = -0.56, 95% CI (-1.421, 0.301), P = 0.203] [27]. Two SRs suggested that rTMS cannot improve depressive symptoms of PD [36, 40]. There is no consensus on the results of SRs in the treatment of PD depressive symptoms with rTMS.

Five SRs used MoCA, MMSE, and cognitive function to evaluate the cognitive impairment of rTMS in the treatment of PD [29–32, 40, 41]. Among the 5 SRs, 2 SRs used MMSE to evaluate cognitive function, and the results showed that rTMS cannot improve the cognitive impairment of PD [40, 41]. Two SRs used MoCA to evaluate cognitive function, and the results showed that rTMS improve the cognitive impairment of PD [29, 40]. In summary, no consistent conclusion has been reached on the treatment of cognitive impairment in PD by rTMS.

## 4. Discussion

In accordance with evidence-based medicine principles, systematic reviews and meta-analyses are considered the most reliable sources of evidence. However, strict adherence to guidelines is essential to minimize bias when addressing research questions. Therefore, high-quality SRs play a critical role in ensuring the validity, clarity, and accurate interpretation of evidence. Despite a growing number of SRs focusing on rTMS for PD, the quality varies and conclusions are inconsistent. This overview aims to comprehensively identify and assess all available evidence regarding the use of rTMS in PD treatment. The research findings and conclusions derived from the inclusion of SRs are summarized.

### 4.1 Summary of the main results

We conducted a comprehensive descriptive analysis of 16 SRs of rTMS for PD, involving 260 original studies. Evidence from the majority of studies suggests that rTMS combined with conventional treatment for PD is more effective than conventional treatment or sham stimulation alone in improving motor symptoms in PD patients and does not cause serious adverse effects. Some of the SRs evidence also suggests that rTMS does not improve PD patients motor symptoms compared to conventional treatment. For non-motor symptoms, such as cognitive function and depressive symptoms, conclusions on rTMS for PD have not been consistent. The methodological quality of the SRs was assessed using the AMSTAR 2 tool, and the methodological quality of the SRs was variable, with 12.5% (2/16) of the SRs rated as high quality, 43.75% (7/16) rated as low quality, and 43.75% (7/16) rated as very low quality. The risk of bias was assessed using ROBIS and 37.5% (6/16) were rated as low risk. The GRADE results suggested that 16.13% (10/62) of the evidence was of moderate quality, 33.87% (21/62) of the evidence was of low quality and 50% (31/62) of the evidence was of very low quality.

According to AMSTAR 2, among the 16 SRs, 12.5% were rated as high quality, and the methodological quality of the remaining (87.5%) SRs was rated as low or very low quality. It is worth noting that in critical item 2, 81.25% (13/16) of SRs did not provide a previous protocol, which means that we cannot guarantee whether the protocol was strictly followed during the research report, thereby increasing the risk of bias. In critical item 4 and 7, 5 (31.25%) reports did not adequately provide search strategies, and 4 (25%) reports did not adequately provide exclusion lists. In addition, some of the SRs were unable to statistically combine results using appropriate methods, did not consider RoB when interpreting results, and did not adequately investigate publication bias. In non-critical items, 68.75% of reviewers performed duplicate literature screening, duplicate data extraction, and described the included studies in sufficient detail. 25% of the reviews did not provide satisfactory explanations and discussions when analyzing heterogeneity. In terms of risk of bias, all SRs rated low risk when assessing relevance. In the second stage, 19.23% of SRs rated high risk when assessing study eligibility criteria, 43.75% of SRs rated high risk when identifying and selecting studies, 31.25% of SRs rated high risk when data collection and study evaluation, and 62.5% of SRs rated high risk when synthesizing and concluding. In the third stage, after comprehensive review, only 37.5% of SRs were rated as low risk. Regarding the evaluation of the quality of the evidence, no high-quality evidence was found, 16.13% of the evidence was of medium quality, 33.87% of low-quality evidence, and 50% of very low-quality evidence. The risk of publication bias, inconsistency and imprecision constituted the most important determinants of evidence downgrading. Significant heterogeneity can lead to inconsistency, while limited sample sizes or too large confidence intervals can lead to imprecision. Notably, although most of the included SRs indicated that rTMS appears to be an effective treatment for PD, most authors did not draw clear conclusions due to the small sample size or low quality of the included trials. Therefore, to determine whether rTMS is beneficial for PD, more high-quality, large-sample RCT studies must be conducted.

rTMS was developed as an extension of TMS, utilizing a consistent stimulation intensity and frequency to target specific brain regions with a series of pulses [43, 44]. This technique is valuable for investigating the functional reorganization of neural networks. rTMS offers the benefits of bidirectional regulation of the cerebral cortex, ease of use, and non-invasiveness. A large number of studies have shown that rTMS can effectively alleviate motor symptoms, cognitive impairment and insomnia symptoms in patients with PD [36, 37, 44]. The therapeutic effects of rTMS on Parkinson’s disease could be linked to a variety of mechanisms. First, rTMS penetrates the skull to the cerebral cortex through magnetic pulses, stimulates the cerebral cortex and peripheral nerves to generate induction currents through the principle of electromagnetic induction, increases the excitability of neurons, prompts the synaptic activation of neurons in the inhibited state, and remodels the damaged neural pathways, which is conducive to the reconstruction of the function of cerebral cortical network, thus improving the symptoms of PD [44, 45]. Second, high-frequency rTMS improves cerebral blood flow, enhancing overall brain excitability and energy metabolism, which can benefit non-motor symptoms of PD, particularly in cognitive domains like language, memory, attention, and executive function [30, 32, 33]. Third, rTMS can induce synaptic structural and functional plasticity changes by influencing synaptic connectivity, neuronal morphology, synaptic gaps, and long-term potentiation (LTP) and long-term depression (LTD), leading to lasting neuroplasticity effects that contribute to long-term improvement in PD symptoms [46, 47]. Lastly, rTMS can activate the phosphorylation process, enhance biological signaling responses, promote neural cell proliferation and differentiation, and regulate the expression and function of various neurotrophic factors, highlighting its potential in neuroprotection and repair [48–50]. In summary, the multi-dimensional action mechanism of rTMS in treating PD is not only reflected in direct neurophysiological regulation, but also includes the enhancement of neuroplasticity and the optimization of the neurotrophic environment. Several studies have shown that rTMS applied to the cerebral cortex can increase oxygen levels in the striatum and thalamus, trigger dopamine release in the striatum, and reduce beta rhythmic neural oscillations in the thalamic nucleus. These findings indicate that the effects of rTMS stimulation may also impact other brain regions that are connected both structurally and functionally [51–53]. The primary motor cortex (M1), dorsolateral prefrontal cortex (DLPFC), and supplementary motor cortex (SMA) are currently the main stimulation target areas for improving motor symptoms of PD. Previous clinical studies have demonstrated that high-frequency rTMS stimulation of the M1 area yields significant improvements in bradykinesia and rigidity [19]. Conversely, low-frequency stimulation of the M1 area does not exhibit a notable impact on motor symptom enhancement. While an increasing number of studies support the efficacy of high-frequency M1 region stimulation in alleviating motor symptoms, its therapeutic benefits remain somewhat limited when compared to dopamine drug therapy [54]. Furthermore, high-frequency rTMS stimulation of the left DLPFC shows promise in ameliorating both motor and non-motor symptoms, particularly depression in PD [55]. Additionally, rTMS stimulation of the SMA has been found to improve freezing of gait [56].

### 4.2 Implications for future study

The included systematic evaluations were assessed using the AMSTAR-2, ROBIS, and GRADE tools, and the results showed limitations in currently published systematic evaluations of rTMS for the treatment of PD, identifying limitations that need to be improved in the future. Only three SRs submitted protocols prior to trial initiation. For the majority of SRs, it is essential to have their study plans pre-registered or published on reputable platforms such as PROSPERO or Cochrane. Additionally, it is crucial to take into account gray literature in the research process. Detailed search curation and exclusion lists should be provided for inclusion and selection of original studies. During search and data extraction, two reviewers should perform literature screening and data extraction, respectively. During the data analysis phase, it is imperative to employ suitable statistical methods to synthesize the findings. The risk of bias (RoB) must be meticulously assessed and factored into the interpretation of the results. Furthermore, a comprehensive examination of publication bias is necessary. In addressing heterogeneity, it is essential to offer a coherent explanation and engage in a thorough discussion to elucidate the observed variability. Many research designs lack proper reporting of blinding procedures, allocation concealment, and randomization methods, leading to increased risks of biases and heterogeneity. The limited number of high-quality randomized controlled trials on rTMS for PD can be attributed to the small number of included studies, low sample size, inconsistent treatment selection, data extraction, and result labeling. However, determining an ideal sample size requires consideration of a variety of statistical and clinical factors, including expected effect size, variability, significance level, and the statistical power of the study. Additionally, ethical and practical recruitment issues need to be considered. We recommend that future researchers consult statisticians when designing large-scale RCTs and use sample size calculation software or methods to determine appropriate sample sizes. Sample sizes used in similar studies, as well as information from clinical trial registries, can be consulted to guide sample size selection. Heterogeneity is further influenced by variations in rTMS parameters, combined therapies, individual differences, mental health, disease severity, and other factors.

### 4.3 Limitation

As an overview of rTMS in the treatment of PD, this study can provide a comprehensive evidence reference for clinical practice. In addition, through the evaluation process of AMSTAR-2, ROBIS and GRADE, it is found that SRs have obvious limitations, which may help guide future high-quality research. This overview has certain limitations. Assessment of quality is a subjective process, and researchers may have their own judgments about each factor. We can only provide a comprehensive and qualitative description of all the data and cannot make a quantitative evaluation. It is recommended that clinical personnel refer to real-life scenarios when using this data for clinical decision-making.

## 5. Conclusion

Based on the collected evidence indicates that repetitive transcranial magnetic stimulation is both effective and safe for enhancing motor function in patients with PD; however, its efficacy on non-motor symptoms remains inconsistent. Although the quality of evidence from some original studies and the methodological rigor of the systematic review diminish the reliability of the conclusions, the overall quality is not high. Nonetheless, based on the current findings, we continue to endorse rTMS as a valuable adjunctive intervention for alleviating PD symptoms. To enhance the accuracy of research and the reliability of conclusions, future studies should address the limitations of existing methodologies, standardize rTMS treatment protocols, conduct comprehensive assessments of potential side effects, and compare the effects of rTMS with those of established treatments.

## Supporting information

S1 Checklist PRISMA 2020 checklist. (DOCX)

S1 Table Search strategy. (DOC)
