# Supplementary material for: Unveiling the efficacy of repetitive transcranial magnetic stimulation in Parkinson’s disease: A comprehensive review of systematic analyses
Source: PLoS One. 2025 Jan 6;20(1):e0313420. doi: 10.1371/journal.pone.0313420 (PMC11703078; doi:10.1371/journal.pone.0313420)
Supplement: S1 Table — (DOC) [file pone.0313420.s002.doc]

***Supplementary Table 1:*** Search strategy.

| **PubMed** |
| --- |
| #1 "Parkinson Disease"[Mesh] |
| #2 "idiopathic parkinsons disease"[Title/Abstract] OR "parkinsons disease idiopathic"[Title/Abstract] OR "parkinson disease idiopathic"[Title/Abstract] OR "parkinsons disease"[Title/Abstract] OR "idiopathic parkinson disease"[Title/Abstract] OR "primary parkinsonism"[Title/Abstract] OR "parkinsonism primary"[Title/Abstract] OR "paralysis agitans"[Title/Abstract] OR "Parkinsonism"[Title/Abstract] |
| #3 #1 OR #2 |
| #4 "Meta-Analysis"[Publication Type] |
| #5 "meta analysis"[Title/Abstract] OR "meta analysis"[Title/Abstract] OR "systematic review"[Title/Abstract] OR "systematic assessment"[Title/Abstract] OR "system evaluation"[Title/Abstract] OR "systematic evaluation"[Title/Abstract] OR "meta analysis as topic"[MeSH Major Topic] |
| #6 #4 OR #5 |
| #7 "Deep Brain Stimulation"[MeSH Terms] |
| #8 (((((Brain Stimulations, Deep[Title/Abstract]) OR (Deep Brain Stimulations[Title/Abstract])) OR (Stimulation, Deep Brain[Title/Abstract])) OR (Stimulations, Deep Brain[Title/Abstract])) OR (Brain Stimulation, Deep[Title/Abstract])) OR (Electrical Stimulation of the Brain[Title/Abstract]) |
| #9 #7 OR #8 |
| #10 #9 AND #6 AND #3 |
| **Embase** |
| #1 ‘meta analysis’/ exp OR ‘meta analysis (topic)’/ exp |
| #2 ‘meta analysis’: ti,ab OR ‘meta analyses’: ti,ab OR ‘meta-analysis’: ti,ab OR ‘meta-analyses’: ti,ab OR meta analysis:ti,ab OR meta analysis: ti,ab OR ‘met-analysis’: ti, ab OR meta analyses: ti, ab OR metanalyses: ti,ab OR ‘clinical trial overview’: ti, ab OR ‘clinical trial overviews’: ti,ab |
| #3 ‘systematic review’/ exp OR ‘systematic review (topic)’/ exp |
| #4 ‘systematic review’: ti,ab OR ‘systematic reviews’: ti, ab |
| #5 #1 OR #2 OR #3 OR #4 |
| #6 ‘repetitive transcranial magnetic stimulation’: ti,ab OR ‘transcranial magnetic stimulation’ : ti,ab OR ‘noninvasive brain stimulation’ : ti,ab |
| #7‘repetitive transcranial magnetic stimulation’/ exp OR ‘transcranial magnetic stimulation’ / exp OR ‘noninvasive brain stimulation’ / exp |
| #8 #6 OR #7 |
| #9 ‘Parkinson Disease’/ exp OR ‘Parkinson’s Disease’ / exp OR ‘Parkinsonism’ / exp |
| #10‘Parkinson Disease’ : ti,ab OR ‘Parkinson’s Disease’ : ti,ab OR ‘Parkinsonism’ : ti,ab |
| #11 #9 OR #10 |
| #12 #5 AND #8 AND #11 |
| **Web of Science** |
| #1 TS =“meta analysis” OR TS =“meta analyses” OR TS =“meta-analysis” OR TS =“meta-analyses” OR TS = “meta-Analysis” OR TS =“metanalysis”OR TS =“ metaanalyses”OR TS =“systematic review” OR TS =“systematic reviews” OR TS =“clinical trial overview” OR TS =“clinical trial overviews” |
| #2 TS =“repetitive transcranial magnetic stimulation” OR TS =“transcranial magnetic stimulation” OR TS =“noninvasive brain stimulation” |
| #3 TS =“Parkinson Disease” OR TS =“Parkinson’s Disease” OR TS =“Parkinsonism” |
| #4 #1 AND #2 AND #3 |
| **The Cochrane Library** |
| #1 MeSH descriptor: [Parkinson’s Disease] explode all trees |
| #2 (Parkinson’s Disease):ab,ti,kw OR (Parkinson Disease):ab,ti,kw |
| #3 #1OR#2 |
| #4 (meta analysis):ab,ti,kw OR (meta analyses):ab,ti,kw OR (meta-analysis):ab,ti,kw OR (meta-analyses):ab,ti,kw OR (meta-Analysis):ab,ti,kw OR (metanalysis):ab,ti,kw OR (metaanalyses):ab,ti,kw OR (systematic review):ab,ti,kw OR (systematic reviews):ab,ti,kw OR (clinical trial overview):ab,ti,kw OR (clinical trial overviews):ab,ti,kw |
| #5 (repetitive transcranial magnetic stimulation):ab,ti,kw OR (transcranial magnetic stimulation):ab,ti,kw OR (noninvasive brain stimulation):ab,ti,kw |
| #6 #3 AND #4 AND #5 |
| **China National Knowledge Infrastructure** |
| ( ( (主题=系统评价 或者 题名=系统评价 或者 v_subject=中英文扩展(系统评价) 或者 title=中英文扩展(系统评价)) 或者 (主题=meta分析 或者 题名=meta分析 或者 v_subject=中英文扩展(meta分析) 或者 title=中英文扩展(meta分析)) ) 或者 ( (主题=荟萃分析 或者 题名=荟萃分析 或者 v_subject=中英文扩展(荟萃分析) 或者 title=中英文扩展(荟萃分析)) 或者 (主题=元分析 或者 题名=元分析 或者 v_subject=中英文扩展(元分析) 或者 title=中英文扩展(元分析)) ) ) 并且 ( ( (主题=重复经颅磁刺激 或者 题名=重复经颅磁刺激 或者 v_subject=中英文扩展(重复经颅磁刺激) 或者 title=中英文扩展(重复经颅磁刺激)) 或者 (主题=经颅磁刺激 或者 题名=经颅磁刺激 或者 v_subject=中英文扩展(经颅磁刺激) 或者 title=中英文扩展(经颅磁刺激)) ) 或者 ( (主题=无创性脑刺激 或者 题名=无创性脑刺激 或者 v_subject=中英文扩展(无创性脑刺激) 或者 title=中英文扩展(无创性脑刺激)) ) ) 并且 ( ( (主题=帕金森病 或者 题名=帕金森 或者 v_subject=中英文扩展(帕金森病) 或者 title=中英文扩展(帕金森氏病)) |
| **Wanfang Database** |
| 检索表达式（中英文扩展&主题词扩展）： 主题:(系统评价+系统综述+meta分析+荟萃分析+元分析)*主题:(经颅磁刺激+重复经颅磁刺激+无创性脑刺激)*主题:(帕金森病+帕金森症+帕金森患者+帕金森综合征) |
| **Chongqing VIP** |
| (U=帕金森病 OR U=帕金森患者 OR U=帕金森综合征) AND (U=重复经颅磁刺激 OR U=经颅磁刺激 OR U=无创性脑刺激 OR U=非侵入性脑刺激) AND (U=荟萃分析 OR U=系统综述 OR U=系统评价 OR U=Meta分析) |

**Supplementary Table 2.** Exclude Articles

| **First Author, year** | **Title** | **Reasons for exclusion** |
| --- | --- | --- |
| Lesenskyj 2018 | Treating refractory depression in Parkinson's disease: a meta-analysis of transcranial magnetic stimulation | Not rTMS |
| Liu 2024 | The effects of transcranial magnetic stimulation for freezing of gait in Parkinson's disease: a systematic review and meta-analysis of randomized controlled trials |
| Fregni 2005 | Non-invasive brain stimulation for Parkinson's disease: a systematic review and meta-analysis of the literature |
| Nardone 2020 | Transcranial magnetic stimulation and gait disturbances in Parkinson's disease: A systematic review |
| Elahi 2009 | Effect of transcranial magnetic stimulation on Parkinson motor function--systematic review of controlled clinical trials |
| Wu 2020 | Transcranial Magnetic Stimulation Alleviates Levodopa-Induced Dyskinesia in Parkinson's Disease and the Related Mechanisms: A Mini-Review |
| Vonloh 2013 | Safety of transcranial magnetic stimulation in Parkinson's disease: a review of the literature |
| Delgado-Alvarado  2020 | Nonpharmacological, nonsurgical treatments for freezing of gait in Parkinson's disease: A systematic review |
| Dong 2023 | Comparative efficacy of transcranial magnetic stimulation on different targets in Parkinson's disease: A Bayesian network meta-analysis | Network meta analysis |
| Hvingelby 2022 | Interventions to improve gait in Parkinson's disease: a systematic review of randomized controlled trials and network meta-analysis |
| Liu 2023 | Comparative motor effectiveness of non-invasive brain stimulation techniques in patients with Parkinson's disease: A network meta-analysis |
| Lawrence 2017 | Cognitive Training and Noninvasive Brain Stimulation for Cognition in Parkinson's Disease: A Meta-analysis | Different TMS methods |
| da Silva Machado 2021 | Multisite non-invasive brain stimulation in Parkinson's disease: A scoping review |
| Zhang 2023 | Effects of non-invasive brain stimulation on walking and balance ability in Parkinson's patients: A systematic review and meta-analysis |
| Madrid 2021 | Non-invasive brain stimulation for Parkinson's disease: Clinical evidence, latest concepts and future goals: A systematic review |
| Wei 2023 | Effects of non-invasive brain stimulation over supplementary motor area in people with Parkinson's disease: a protocol for a systematic review and meta-analysis of randomised controlled trials | Not SRs |
| Randver 2018 | Repetitive transcranial magnetic stimulation of the dorsolateral prefrontal cortex to alleviate depression and cognitive impairment associated with Parkinson's disease: A review and clinical implications |
